# The Relationship between Osteoporosis and Osteoarthritis of the Knee: A Report of 2 Cases with Suspected Osteonecrosis

**DOI:** 10.1155/2014/514058

**Published:** 2014-06-17

**Authors:** Akira Horikawa, Naohisa Miyakoshi, Yoichi Shimada, Hiroyuki Kodama

**Affiliations:** ^1^South Akita Orthopedic Clinic, Seiwakai, 96-2 Kaidousita, Syowa-Ookubo, Katagami 018-1401, Japan; ^2^Department of Orthopedic Surgery, Akita University Graduate School of Medicine, 1-1-1 Hondo, Akita 010-8543, Japan

## Abstract

Knee specimens of two osteoporotic patients who underwent unilateral knee arthroplasty for suspected osteonecrosis of the knee were examined histologically. Preoperative findings of magnetic resonance images in both patients were consistent with the diagnosis of osteonecrosis of the medial femoral condyles, although plain X-rays showed minimal degenerative changes. In both patients, preoperative bone mineral densities of the femoral condyle and proximal tibia of the affected side were lower than those of the unaffected side. Pathological examination of the resected femoral condyle and proximal tibia showed almost intact joint cartilage, healing of the collapsed subchondral bone, and significant trabecular bone loss. Histologically, no evidence of osteonecrosis, including empty lacunae of the trabecular bone, was observed. These findings indicated that subchondral bone collapse caused by osteoporosis, but not osteonecrosis, initiated the osteoarthritic change of the affected knee. This report emphasizes that there may be cases of progressive local osteoarthritis caused by fracture of subchondral bone because of osteoporosis.

## 1. Introduction

There are many theories about the cause of osteoarthritis. However, because there are two major reports about the relationship between osteoporosis and osteoarthritis, it remains controversial. The first is that reduced bone mineral density (BMD) of the subchondral bone is observed in the early stage of osteoarthritis, although subchondral bone sclerosis and higher BMD are seen radiologically, which might indeed predict development of a cartilage defect [1]. The second is that females with a high BMD have a high incidence of osteoarthritis, while the progression of osteoarthritis is relatively slow compared to that seen with a low BMD [2]. Moreover, there are numerous theories in relation to the following inconsistencies: the risk of osteoarthritis rises in proportion to the increase in BMD, while it decreases with the accumulation of BMD [3–6]. Although there are many multifactorial pathogeneses of spontaneous osteonecrosis of the knee (SONK), one theory advocated that SONK was induced by subchondral insufficiency fracture that may be associated with osteoporosis [7], and the incidence rate was assumed to be about 10% in osteoarthritis of the knee in the initial phase, especially in association with SONK [8]. In this paper, we present two cases with osteoarthritis of the knee initially diagnosed as SONK. Based on our experience using magnetic resonance imaging and pathological examination, it appears that our cases are inconsistent with the hypothesis that low BMD is a risk factor with microfracture that might lead to permanent osteonecrosis. In fact, the present cases suggest that the initial radiological phase of SONK may be curable, but resultant irregularity of joint surface induced osteoarthritis.

## 2. Case Report

### 2.1. Case  1

**Figure 1:**
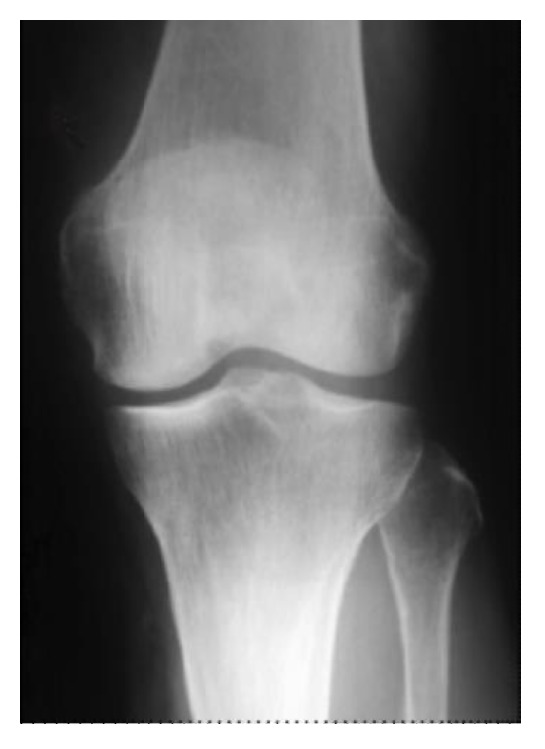
Case 1. Preoperative plain radiograph reveals no degenerative changes, suggesting Kellgren-Lawrence grade 1.

**Figure 2:**
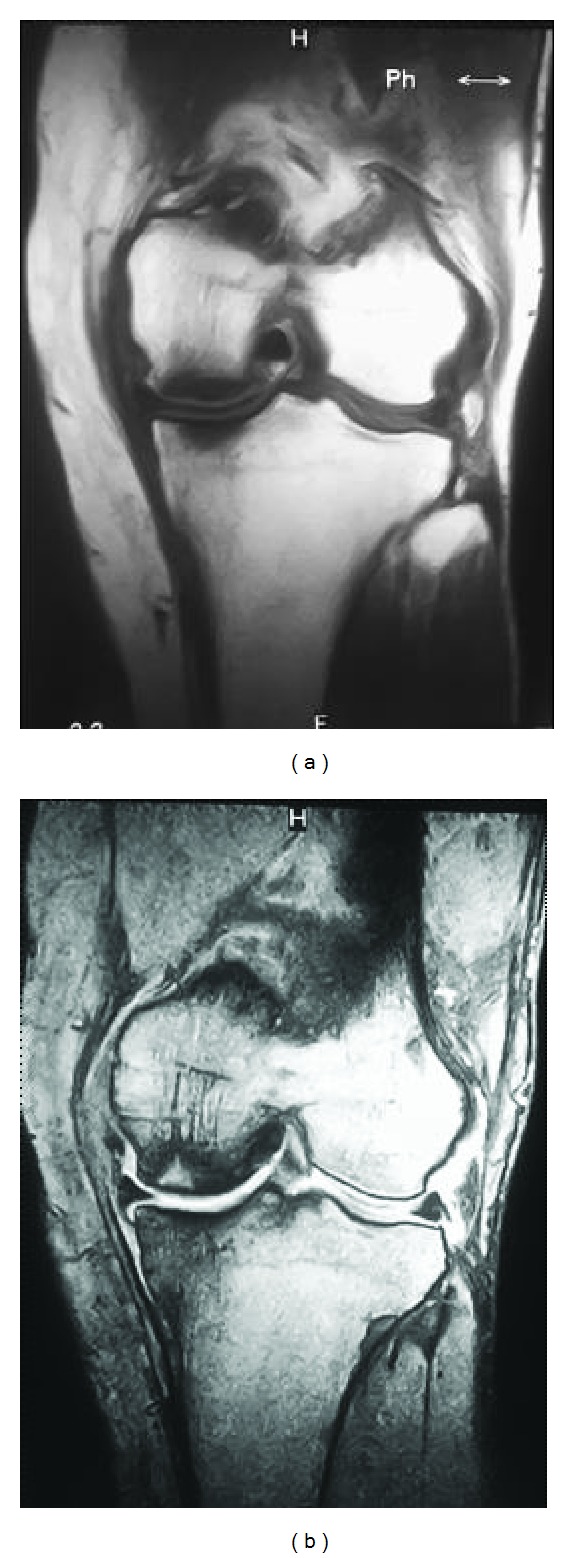
Case 1. Preoperative magnetic resonance images of the affected knee. T1-weighted image shows low signal intensity of the medial femoral condyle and tibial plateau (a). T2-weighted image shows high intensity in the central area of the femoral lesion and the surrounding low-intensity area (b).

A 73-year-old woman complained of left knee pain without a history of trauma or any systemic disorder that might have caused joint pain. Physical examination revealed tenderness in the medial knee joint. In addition, plain radiographs revealed no degenerative changes, suggesting Kellgren-Lawrence grade 1 (Figure 1). The femorotibial angle was 173° on standing. Laboratory findings were within normal limits. Areal BMD measurement was performed at the proximal femur using dual energy X-ray absorptiometry (DXA) (Hologic QDR Discovery W type, Toyo Medic., Tokyo, Japan) and suggested a diagnosis of osteoporosis, with a* T*-score of 59%. Magnetic resonance imaging (MRI) showed low signal intensity on T1-weighted imaging (Figure 2(a)), while the intensity was high in the center and low in the surrounding area of the medial femoral condyle on T2-weighted imaging, suggesting osteonecrosis (Figure 2(b)).

**Figure 3:**
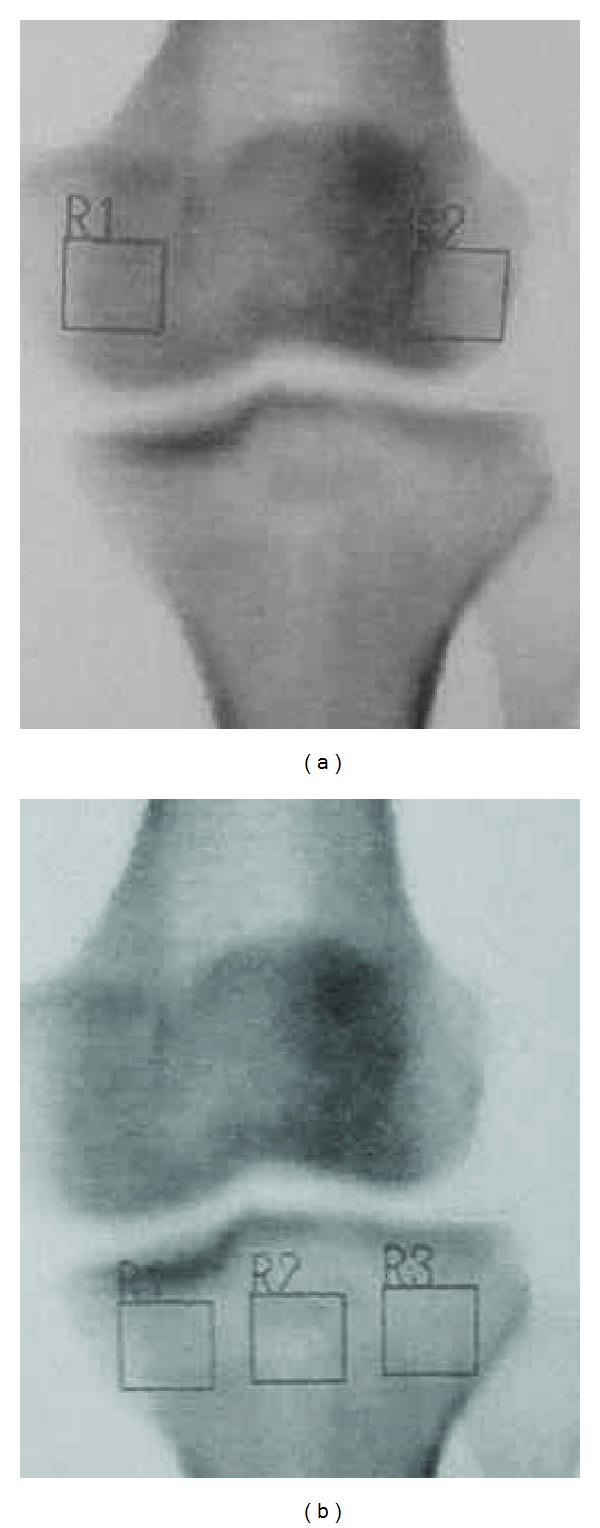
An example of the measurement areas of the bone mineral density of the knee condyles.

To investigate the cause of the relationship between osteoporosis and osteoarthritis, further examinations were performed using DXA of the femoral condyle and the proximal part of the tibia. Localized BMD measurements of the knee condyles were performed with the patient in the supine position on the scanning table. In the tibial condyles, three square regions of interest were marked on the frontal view (Figure 3). A line extending to the lateral and medial edges of the proximal tibia was divided into three equal lengths, and the regions of interest (ROI) were marked underneath. Thus, three tibial condyle BMDs were calculated for the tibia. Moreover, the medial and lateral femoral condyle BMDs in the square regions of interest of the same size were also calculated using the same methods.

The BMDs of the femoral condyle and the tibial plateau were lower on the affected side than on the healthy side (Table 1), showing that the BMD of the affected knee joint was lower than that of the unaffected knee joint.

**Figure 4:**
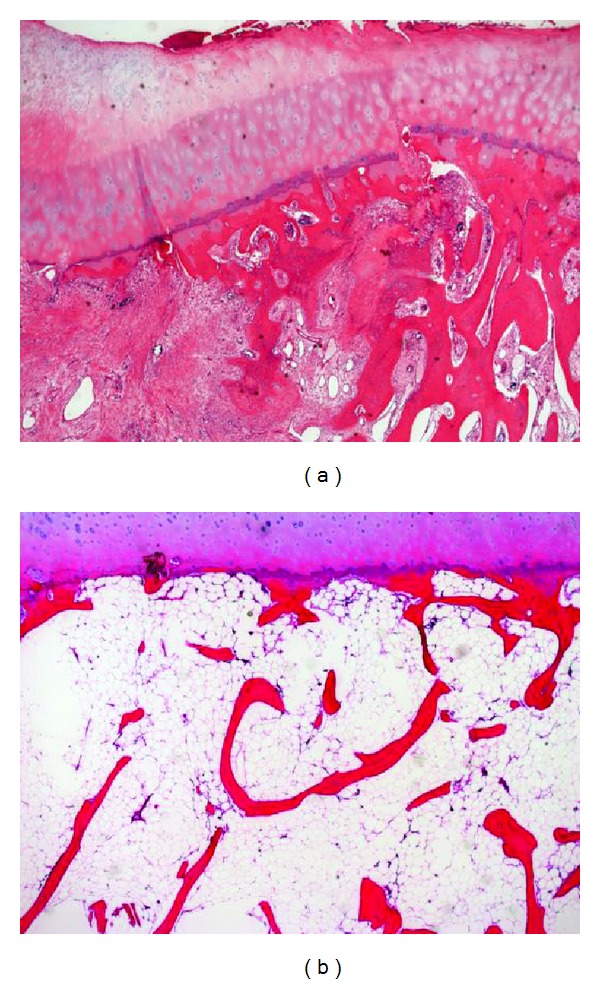
Photomicrographs of the femoral condyle obtained from the surgical site in Case 1. A specimen obtained from the central area of the lesion shows less destructive joint cartilage, healing of the subchondral bone, and migration of the fibrous tissue into the subchondral area (a). A specimen from the surrounding area of the lesion shows normal joint cartilage, thinning of trabecular bone, and lesser trabecular connectivity, suggesting osteoporosis of the subchondral area (b). Hematoxylin and eosin stain. Original magnification ×40.

Unilateral knee arthroplasty was performed. Pathological changes of the femoral component, the suspected osteonecrotic area, were examined. On pathological examination, the superficial cartilage layer was intact, and there were some repaired areas after fracture of subchondral bone with accompanying migration of fibrous tissue (Figure 4(a)). Subchondral bone adjacent to the collapsed area showed thinning of the trabecular bone and less trabecular connectivity (Figure 4(b)).

### 2.2. Case  2

**Figure 5:**
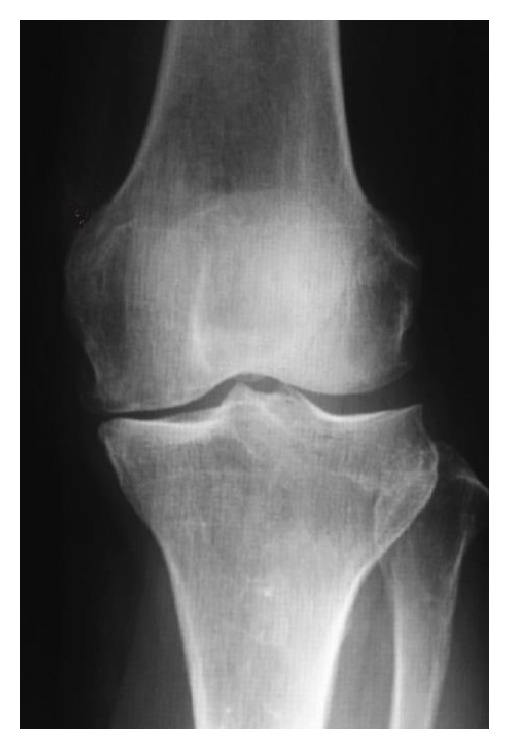
Preoperative plain radiograph of Case 2 shows slight degenerative changes, suggesting Kellgren-Lawrence grade 2.

**Figure 6:**
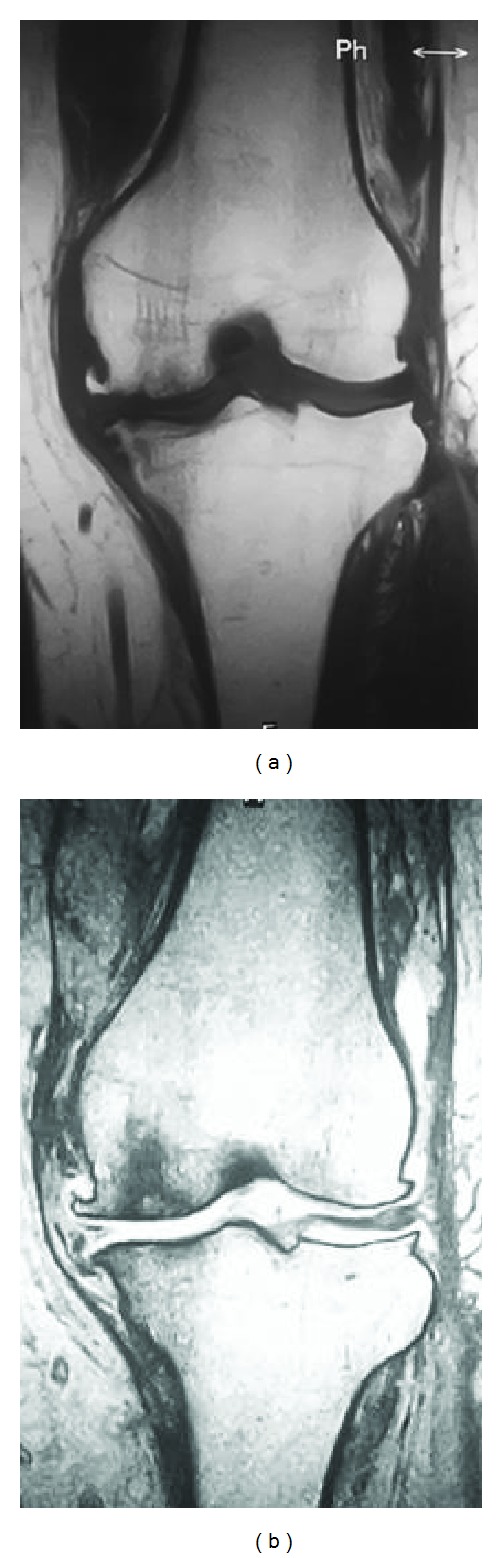
Preoperative magnetic resonance images of the affected knee in Case 2. T1-weighted image shows low signal intensity on the medial femoral condyle and tibial plateau (a). T2-weighted image shows high intensity in the central area of the femoral lesion and the surrounding low-intensity area (b).

A 70-year-old woman was diagnosed with osteoarthritis of the left knee one year earlier. She received physical therapy but complained of sudden acute pain. Physical examination revealed tenderness of the medial knee joint and no effusion, and plain radiographs demonstrated degenerative changes, which implied Kellgren-Lawrence grade 2 (Figure 5). The femorotibial angle was 178° on standing. Laboratory findings were within normal limits. BMD of the proximal femur was 67%, indicating osteoporosis. MRI showed intensity changes similar to those of Case 1 (Figures 6(a) and 6(b)).

The BMDs of the femoral condyle and the tibial plateau were measured with the above-mentioned technique, and the BMDs were lower on the affected side than on the healthy side (Table 1).

This patient also underwent unilateral knee arthroplasty. Pathological findings of the femoral condyle of this case were consistent with those of Case 1. No histological evidence of osteonecrosis was seen, but healed subchondral bone collapse and osteoporosis were observed.

At the latest postoperative follow-up at 2 years, both patients had no complaints of knee pain, and radiographic examinations showed no abnormal findings.

## 3. Discussion

At present, many perceive a strong relationship between BMD and osteoarthritis of the knee, while others regard them as distinct. Research reports thus far have been conflicting and inconclusive. Some reports have shown that the risk of osteoarthritis rises in proportion to the increase of BMD of the lumbar spine [3, 4]. Conversely, other reports have shown the risk of osteoarthritis rising in proportion to the decrease of BMD of the femoral neck [5, 6]. The former advocated that high BMD overloaded the knee joint surface, causing joint cartilage damage that induces joint space narrowing as if it were severe degeneration. The latter suggested that subchondral bone of osteoporotic patients was exposed to direct mechanical stress via joint cartilage, causing the cancellous bone surrounding the subchondral bone to change, implying microfracture and osteonecrosis [9] and leading to promotion of valgus deformity of the knee, such as through joint surface decompression [10]. Furthermore, it is also thought that SONK is a faulty union of the bone due to ischemic change [11]. Whether these theories are correct remains unclear.

Although a clear consensus has yet to be reached, the present cases had low BMDs and might have had microfractures. Therefore, the focus of our cases was on the low BMD and the radiological and pathological findings. These two cases were diagnosed as osteonecrosis of the medial femoral condyle based on the preoperative imaging and clinical findings. However, the histological findings of the surgical specimens revealed that these lesions were subchondral bone collapse in the femoral medial condyle caused by severe osteoporosis and the lesions were histologically healed with irregularity of the joint surface. That means that fragility of subchondral bone in the medial femoral condyle might have a close relationship with the imaging diagnosis of SONK.

Karvonen et al. [12] suggested that BMD of the subchondral bone in middle-grade osteoarthritis of the knee is lower than that of the surrounding joint surface in osteoarthritic knees, which implies that the subchondral bone area in the initial phase of osteoarthritis of the knee is fragile with mechanical stress, such as weight-bearing. Moreover, Goerres et al. [6] noted that osteoarthritis of the knee could lead to detection of SONK, which means idiopathic bone necrosis on MRI in Kellgren-Lawrence stages 1-2. Kesemenli et al. [13] noted that SONK is regarded as a microfracture. Karachalios et al. [8] also noted that osteoporotic joint subchondral bone can lead to bone fragility, causing repeated microfractures, and its accumulation finally leads to osteonecrosis.

Considering these findings and theories, we assume that the osteoarthritis of the knee with sudden onset was due to collapse of subchondral bone with loss of BMD. Although there have been some reports that osteonecrosis was the cause of osteoarthritis in the light of radiological findings, as a matter of fact, there might exist cases of progressive local osteoarthritis that were caused by fracture of subchondral bone because of osteoporosis.

In other words, osteoporotic patients who complain of knee pain might develop microfractures of the medial femoral condyle that contribute to SONK. Therefore, it is important for orthopedic surgeons to screen for the initial phases of SONK and to treat it appropriately as soon as possible. Presumably, there should be cases of SONK on radiological examination that do not show necrotic lesions on histological examination.

In summary, two osteoporotic patients who had low BMD of the femoral condyle and tibial plateau and a preoperative imaging diagnosis of SONK were described. They underwent surgery, and the surgical specimens showed healing of subchondral bone collapse and significant osteoporosis. BMD data of the affected knees and histological examinations following surgery strongly supported the view that local bone loss had some relationship with the imaging diagnosis of SONK.

## Figures and Tables

**Table 1:** Bone mineral density (g/cm^2^) in the femoral epicondyle and tibial plateau.

	Case 1	Case 2
Femoral condyle		
Affected side	0.288	0.378
Healthy side	0.502	0.677
Tibial plateau		
Affected side	0.103	1.060
Healthy side	0.167	1.205
